# National prevalence of smoking among adolescents at tobacco tax increase and COVID-19 pandemic in South Korea, 2005–2022

**DOI:** 10.1038/s41598-024-58446-4

**Published:** 2024-04-03

**Authors:** Seohyun Hong, Selin Woo, Seokjun Kim, Jaeyu Park, Myeongcheol Lee, Sunyoung Kim, Ai Koyanagi, Lee Smith, Min Seo Kim, Guillermo F. López Sánchez, Elena Dragioti, Masoud Rahmati, Guillaume Fond, Laurent Boyer, Jiyeon Oh, Hojae Lee, Dong Keon Yon

**Affiliations:** 1 Department of Medicine, Kyung Hee University College of Medicine, Seoul, South Korea; 2 Center for Digital Health, Medical Science Research Institute, Kyung Hee University College of Medicine, Seoul, South Korea; 3 Department of Regulatory Science, Kyung Hee University, Seoul, South Korea; 4 Department of Family Medicine, Kyung Hee University Medical Center, Kyung Hee University College of Medicine, Seoul, South Korea; 5 Research and Development Unit, Parc Sanitari Sant Joan de Deu, Barcelona, Spain; 6 Centre for Health, Performance and Wellbeing, Anglia Ruskin University, Cambridge, UK; 7 Cardiovascular Disease Initiative, Broad Institute of MIT and Harvard, Cambridge, MA USA; 8 Division of Preventive Medicine and Public Health, Department of Public Health Sciences, School of Medicine, University of Murcia, Murcia, Spain; 9 Pain and Rehabilitation Centre, and Department of Medical and Health Sciences, Linköping University, Linköping, Sweden; 10 Research Laboratory Psychology of Patients, Families, and Health Professionals, Department of Nursing, School of Health Sciences, University of Ioannina, Ioannina, Greece; 11 Department of Physical Education and Sport Sciences, Faculty of Literature and Human Sciences, Lorestan University, Khoramabad, Iran; 12 Department of Physical Education and Sport Sciences, Faculty of Literature and Humanities, Vali-e-Asr University of Rafsanjan, Rafsanjan, Iran; 13 CEReSS-Health Service Research and Quality of Life Center, Assistance Publique Des Hôpitaux de Marseille, Aix-Marseille University, Marseille, France; 14 Department of Pediatrics, Kyung Hee University Medical Center, Kyung Hee University College of Medicine, 23 Kyungheedae-ro, Dongdaemun-gu, Seoul, 02447 South Korea

**Keywords:** Adolescent, COVID-19, Daily smokers, Ever smokers, Interrupted time series, ARIMA, Smoking, Tobacco tax, Health care, Health policy, Public health

## Abstract

Prior research has predominantly focused on the overall effects of the tobacco tax increase and the COVID-19 pandemic on adolescent smoking behavior. However, there is a need to examine both the immediate and sustained associations of these two factors on subgroups of adolescents, employing an interrupted time-series model. We aimed to investigate the immediate and sustained association of tobacco tax increase and the COVID-19 pandemic on adolescent smoking prevalence. This study utilized data from the Korea Youth Risk Behavior Web-Based Survey to analyze the prevalence of current smoking among all participants (CSP) and the prevalence of daily smoking among current smokers (DSP) of Korean adolescents (n = 1,159,995; mean, age 14.99; male 51.5%) over 18 years from 2005 to 2022. The study examined 18-year trends in CSP and DSP among Korean adolescents, emphasizing the influences of the 2015 tobacco tax increase and the COVID-19 pandemic, using β coefficients and their differences (β_diff_) from an interrupted time-series ARIMA model. While CSP exhibited a decreasing trend, DSP exhibited an increasing trend. Tobacco tax increase was associated with both the short and long terms in smoking prevalence, however, the short-term association on prevalence (CSP, − 3.076 [95% CI, − 3.707 to − 2.445]; DSP, − 4.112 [95% CI, − 6.488 to − 1.735]) was stronger. The pandemic was associated with an immediate increase in DSP (9.345 [95% CI, 5.285–13.406]). These effects were strongest among adolescents from low economic status and those exposed to familial secondhand smoking. Supportive programs for adolescents in low-income families will help overcome the effects associated with the pandemic. As a tobacco tax increase was associated with a reduction in smoking prevalence, this could be one method to overcome the effects of the pandemic.

## Introduction

Adolescent smoking is a persistent and pressing public health concern, with implications that extend far beyond individual health to encompass societal well-being. According to the World Health Organization (WHO), adolescents are more susceptible to nicotine addiction than adults, making early intervention crucial in addressing this issue effectively^1^. Recognizing the gravity of tobacco exposure during adolescence, many countries have implemented various tobacco control strategies, such as smoking cessation programs and tobacco tax increases, to mitigate adolescent smoking^2,3^. Among these strategies, the tobacco tax increase has gained prominence as an effective approach, as it is expected to reduce tobacco demand by impacting the supply–demand curve^4,5^. Previous research has demonstrated that higher tobacco prices, primarily resulting from tax increases, can reduce the initiation and prevalence of smoking among adolescents^4^.

Several factors, including sex, socioeconomic status, academic performance, stress conditions, and residence, have been identified as influencing adolescent smoking behaviors^6^. Moreover, the emergence of the COVID-19 pandemic has introduced additional complexities, potentially exacerbating adolescent smoking due to factors like social isolation, anxiety, and stress^7^. Therefore, it is imperative to comprehensively understand the impact of tobacco tax increases and the COVID-19 pandemic on both short-term and long-term trends in adolescent smoking prevalence. However, the specific impacts of the COVID-19 pandemic on adolescent smoking, particularly in conjunction with increased tobacco taxes, remain underexplored.

While previous studies have examined the prevalence of adolescent smoking, there remains a notable gap in characterizing the rate of adolescent daily smokers among current smokers based on stratified groups. Addressing this gap is significant as it can provide crucial insights into the intensity of smoking and the risk factors associated with daily smoking behavior. Given that daily smoking is associated with a higher risk of developing smoking-related issues^8,9^, understanding the prevalence of daily smoking among current smokers is essential for informing the development of targeted interventions and control policies aimed at reducing daily smoking behaviors. Preventive strategies may include educational campaigns tailored to different demographic groups, school-based interventions that focus on building peer support and resilience, community outreach programs that address socioeconomic factors that influence smoking behavior, and policy initiatives aimed at restricting access to tobacco products and promoting smoke-free environments^10^. Thus, this study employs interrupted time-series analysis (ITSA) to scrutinize the pragmatic short-term and long-term effectiveness of the tobacco tax increase and the COVID-19 pandemic on adolescent smoking prevalence^11,12^.

This study seeks to illuminate the intricate relationship between tobacco tax policies, the COVID-19 pandemic, and the multifaceted factors influencing adolescent smoking behavior. Through these insights, we aspire to contribute to evidence-based policies and interventions that can effectively mitigate the adverse effects of smoking on the health and well-being of our youth.

## Methods

### Sample selection and data collection

Data were collected and organized between 2005 and 2022 by the Korea Youth Risk Behavior Web-based Survey (KYRBS), which is an anonymous survey conducted annually to understand the health status of Korean adolescents (13–18 years old)^13–15^. The KYRBS investigates health risk behaviors among middle and high school students, such as socioeconomic conditions, physical activity status, weight control efforts, dietary behavior, drinking and smoking status, substance abuse, and mental health. The survey was conducted with government support and utilized surveys facilitated under the auspices of the Korea Disease Control and Prevention Agency (KDCA). To ensure representative sampling, all middle and high schools across the country were stratified based on urban, suburban, and rural classifications. The ratio of middle schools to high schools in the sample was set at 1:1. Initially, a total of 5 middle schools and 5 high schools were selected. Subsequently, sample schools were allocated based on urban/rural locations and sex compositions, considering female-only, male-only, and mixed-sex schools. Finally, in the nationwide study, a total of 800 sample schools were selected, and to determine the sample classes, the class information of these sample schools was registered^13,14^. Subsequently, the investigation was conducted targeting these sample classes. Adolescents were recruited by web-based survey at their individual schools. Across 18 years of the survey, a total of 1,197,028 answers were collected. However, we excluded those with missing values and responses, and finally, a total of 1,159,995 adolescents (mean age, 14.99; male 51.5%) were included in the final dataset.

The KYRBS data were anonymous and the study protocol was approved by the KDCA and Institutional Review Board of Kyung Hee University (KHUH 2022-06-042). All participants provided written informed consent. Our study adhered to the tenets of the Declaration of Helsinki.

### Covariate definitions

We defined current smoking prevalence (CSP) as the percentage of adolescent current smokers among all adolescents and daily smoking prevalence (DSP) as the percentage of adolescent daily smokers among currently smoking adolescents^16^. Recognizing that daily smokers are more susceptible to negative health consequences compared to non-daily smokers, an increase in DSP indicates even more sever health issues among adolescents than merely suggesting a rise in the number of current smokers. The adolescent population was further stratified based on sex, grade (middle school and high school), body mass index (BMI [kg/m^2^], underweight [< 18.5], normal [18.5–22.9], and overweight [≥ 23])^17^, socioeconomic status (low, average, and high), academic performance (low, average, and high), residence (rural, suburban, and urban)^5,18–20^, educational level of parents (middle school or lower, high school, and college or higher), indirect smoking within family (no and yes), stress conditions (low, average, and high), suicidal ideation within a year (no and yes), and alcohol consumption last month (no and yes) for further stratified analysis. BMI was calculated from self-reported height and weight (Table 1).

**Table 1:** Demographic characteristics of participating adolescent in the KYRBS, 2005–2022 (total n = 1,159,995).

Variables	Crude			
	Overall	Pre-tax-increase period	Post-tax-increase period	Pandemic
Total. number	1,159,995	698,279	304,284	157,432
Sex, number (%)				
Male	597,633 (51.5)	360,034 (51.6)	156,390 (51.4)	81,209 (51.6)
Female	562,362 (48.5)	338,245 (48.4)	147,894 (48.6)	76,223 (48.4)
Grade, number (%)				
Middle school	686,364 (59.2)	415,582 (59.5)	179,164 (58.9)	91,618 (58.2)
High school	473,631 (40.8)	282,697 (40.5)	125,120 (41.1)	65,814 (41.8)
BMI^a^, number (%)				
Underweight	287,456 (24.8)	184,371 (26.4)	68,219 (22.4)	34,866 (22.1)
Normal	604,381 (52.1)	374,625 (53.6)	155,964 (51.3)	73,792 (46.9)
Overweight	268,158 (23.1)	139,283 (19.9)	80,101 (26.3)	48,774 (31.0)
Economic status, number (%)				
Low	212,608 (18.3)	150,548 (21.6)	43,714 (14.4)	18,346 (11.7)
Average	574,250 (49.5)	354,956 (50.8)	143,543 (47.2)	75,751 (48.1)
High	373,137 (32.2)	192,775 (27.6)	117,027 (38.5)	63,335 (40.2)
Academic performance, number (%)				
Low	391,347 (33.7)	242,980 (34.8)	98,369 (32.3)	49,998 (31.8)
Average	352,963 (30.4)	216,810 (31.0)	88,222 (29.0)	47,931 (30.4)
High	415,685 (35.8)	238,489 (34.2)	117,693 (38.7)	59,503 (37.8)
Residence, number (%)				
Rural	123,547 (10.7)	88,240 (12.6)	23,451 (7.7)	11,856 (7.5)
Suburban	500,382 (43.1)	277,119 (39.7)	145,754 (47.9)	77,509 (49.2)
Urban	536,066 (46.2)	332,920 (47.7)	135,079 (44.4)	68,067 (43.2)
Educational level of parents, number (%)				
Middle school or lower	32,975 (2.8)	28,933 (4.1)	3077 (1.0)	965 (0.6)
High school	362,510 (31.3)	268,992 (38.5)	70,604 (23.2)	22,914 (14.6)
College or higher	570,247 (49.2)	319,766 (45.8)	169,596 (55.7)	80,885 (51.4)
Missing values	194,263 (16.7)	80,588 (11.6)	61,007 (20.1)	52,668 (33.4)
Familial secondhand smoking, number (%)				
No	722,871 (62.3)	386,051 (55.3)	217,987 (71.6)	118,833 (75.5)
Yes	381,303 (32.9)	256,407 (36.7)	86,297 (28.4)	38,599 (24.5)
Stress condition, number (%)				
Low	202,756 (17.5)	111,835 (16.0)	60,535 (19.9)	30,386 (19.3)
Average	482,898 (41.6)	285,829 (40.9)	129,303 (42.5)	67,766 (43.0)
High	474,341 (40.9)	300,615 (43.1)	114,446 (37.6)	59,280 (37.7)
Suicidal ideation within a year, number (%)				
No	970,606 (83.7)	565,046 (80.9)	267,570 (87.9)	137,990 (87.7)
Yes	189,389 (16.3)	133,233 (19.1)	36,714 (12.1)	19,442 (12.3)
Alcohol consumption within a month, number (%)				
No	323,294 (27.9)	215,430 (30.9)	73,219 (24.1)	34,645 (22.0)
Yes	836,701 (72.1)	482,849 (69.1)	231,065 (75.9)	122,787 (78.0)

BMI, body mass index; KYRBS, Korea Youth Risk Behavior web-based Survey.

^a^BMI was divided into three groups according to the KDCA: underweight (< 18.5 kg/m^2^); normal (18.5 to 22.9 kg/m^2^); and overweight (≥ 23.0 kg/m^2^).

### End point

This study aimed to investigate the association of the COVID-19 pandemic with the trend of CSP and DSP across 18 years. To achieve this objective, a comparative evaluation of adolescent smoking was undertaken by contrasting the trends during the pandemic (2020–2022) with the pre-pandemic (2005–2019)^14^. However, a discernible inflection point around 2015, potentially caused by the impact of the increase in tobacco tax in South Korea, necessitated a partitioning of the pre-pandemic into two distinct sub-periods: the pre-tax-increase period (2005–2014) and the post-tax-increase period (2015–2019)^21^. In South Korea, a significant increase in tobacco tax occurred in 2015 within the time period 2005–2022. Prior to 2015, the tobacco tax was 1565 South Korean won (₩), with a tobacco price of 2500₩, after 2015, the tax increased to 3318₩, resulting in a tobacco price of 4500₩ (one US dollar [$] is approximately equivalent to 1300 South Korean won [₩]). Therefore, the year 2015 was set as the time point of interruption, partitioning the pre/post-tax-increase-period. This dissection facilitates a more lucid analysis of the influence of the COVID-19 pandemic on adolescent smoking.

### Statistical analysis

In order to investigate long-term trends of CSP and DSP, data from KYRBS between 2005 and 2022 were analyzed. We conducted weighted complex sampling analysis to represent the nationwide population of adolescents, and a weighted linear regression model was applied to calculate weighted β coefficients with 95% confidence intervals (CIs)^22^. In this analysis, the β coefficients indicates the slope of the prevalence (CSP and DSP) over time. Specifically, these coefficients quantify the rate of change in the prevalence of smoking behavior per year. Interrupted time series analysis (ITSA) was used to assess the impact of the tobacco tax increase and the COVID-19 pandemic on adolescent smoking prevalence. ITSA enables the estimation of both immediate effects, which represent the difference between predicted values immediately following the intervention, and sustained effects, which represent the change in trend following the intervention^11^. By accounting for temporal trends and confounding variables, ITSA provides estimation into both short-term changes following policy implementation and long-term trends^23^. Therefore, ITSA allows us to examine the immediate and sustained effects of the tobacco tax increase and the COVID-19 pandemic on adolescent smoking behavior, making it ideal for our study. For ITSA, ARIMA model was constructed based on the following Eq. (1):

1 $$Y = \alpha_{0} + \alpha_{1} T + \alpha_{2} \times 1\left\{ {T \ge \tilde{T}} \right\} + \alpha_{3} \left( {T - \tilde{T}} \right) \times 1\left\{ {T \ge \tilde{T}} \right\} + \alpha_{4} \times 1\left\{ {T \ge \ddot{T}} \right\} + \alpha_{5} \left( {T - \ddot{T}} \right) \times 1\left\{ {T \ge \ddot{T}} \right\} + \varepsilon$$

$Y$ stands for outcomes, which are CSP and DSP that are weighted and stratified. $T$ is a running counter indicating years passed from the start of the observational period. $\tilde{T}$ is a breakpoint specified in the first year of the post-tax-increase period (2015), and $\ddot{T}$ is a breakpoint specified in the first year of the pandemic (2020). According to the model, α_0_ is the mean outcome at the beginning of the observation period; α_1_ is the slope of $Y$ before the first breakpoint; α_2_ shows whether the mean value of $Y$ changed immediately after the first breakpoint; and α_3_ is the difference in the slope of $Y$ before compared with after the first breakpoint. Hence, α_1_ + α_3_ will show the slope of $Y$ after the first breakpoint. α_4_ and α_5_ have similar meanings with α_2_ and α_3_, only that they show the effect of the second breakpoint. $\varepsilon$ shows error. The β difference (β_diff_) was analyzed by α_3_ and α_5,_ representing the difference of trend among pre-price-rise period, post-price-rise period, and the pandemic. Therefore, our model estimates two types of effect, the sustained effect and the immediate effect. The sustained effect is estimated by the change in the level of these slopes at the intervention year. The immediate effect is the difference between the expected value predicted by the model that included the interruption and the expected value with no intervention, at the intervention year.

All analyses in the study were performed via SPSS (version 29.0; IBM Corp., Armonk, NY, USA) and R software (version 4.3.1; R Foundation, Vienna, Austria). Statistical significance was set when a two-sided P-value was less than 0.05^24–27^.

## Results

Crude data comprising a total of 1,159,995 Korean adolescents were collected by KYRBS from 2005 to 2022, encompassing 597,633 men (crude, 51.5%; weighted, 52.4%; 95% CI 51.7–53.1%). Within the pandemic (2020–2022), 157,432 adolescents participated, constituting the entire population of 7,658,833 adolescents (95% CI 7,514,716–7,802,950) (Table 1).

**Figure 1:**
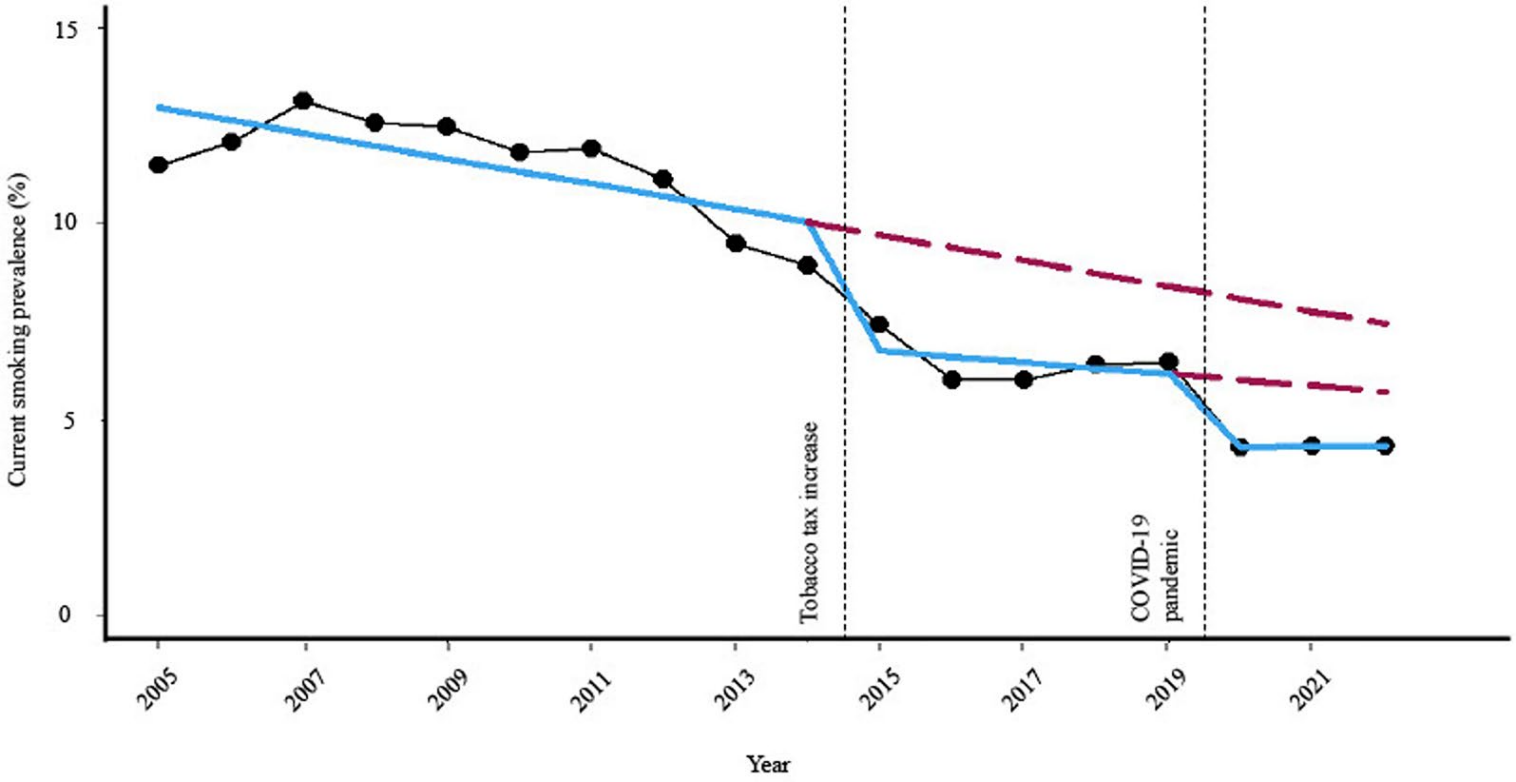
Annual-level overall CSP of adolescents in South Korea between 2005 and 2022, weighted %, in the KYRBS. Data are as observed (measured, black line; trend, blue line) from an interrupted time-series analysis. Dashed red line shows predicted prevalence if the tobacco tax increase and the COVID-19 pandemic didn’t occur.

According to Table 2, CSP decreased during the study period (pre-tax-increase period: 11.529 [95% CI 11.329–11.728], post-tax-increase period: 6.472 [95% CI 6.265–6.679], and pandemic: 4.325 [95% CI 4.143–4.508]). According to Table S1, DSP increased during the study period (pre-tax-increase period: 47.251 [95% CI 46.661–47.841]; post-tax-increase period: 47.667 [95% CI 46.710–48.624]) to the pandemic (50.372 [95% CI 48.968–51.777]). Figure 1 and Figure S1 depict the trend in CSP (Fig. 1) and DSP (Figure S1) among adolescents, using ITSA to examine the impacts of two interruptions: the tobacco tax increase and the COVID-19 pandemic.

**Table 2:** The trend of the current smoking prevalence of adolescents, β-coefficients before and after tobacco tax increase and the COVID-19 pandemic, weighted % (95% CI), in the KYRBS.

CSP	Prevalence, weighted % (95% CI)			Trend analysis, β (95% CI)^a^		
	Pre-tax increase-period (2005–2014)	Post-tax increase-period (2015–2019)	Pandemic (2020–2022)	Trend of the pre-tax-increase-period (2005–2014)	Trend of the post-tax-increase-period (2015–2019)	Trend of the pandemic (2020–2022)
Overall	11.529 (11.329 to 11.728)	6.472 (6.265 to 6.679)	4.325 (4.143 to 4.508)	−** 0.335 (**−** 0.400 to **−** 0.270)**	−** 0.166 (**−** 0.317 to **−** 0.015)**	0.018 (− 0.213 to 0.250)
Sex						
Male	15.806 (15.524 to 16.087)	9.671 (9.372 to 9.970)	5.950 (5.669 to 6.232)	−** 0.096 (**−** 0.190 to **−** 0.001)**	−** 0.559 (**−** 0.778 to **−** 0.341)**	0.060 (− 0.297 to 0.416)
Female	6.769 (6.585 to 6.954)	2.997 (2.842 to 3.152)	2.578 (2.417 to 2.740)	−** 0.586 (**−** 0.646 to **−** 0.527)**	**0.266 (0.149 to 0.383)**	− 0.017 (− 0.215 to 0.180)
Grade						
Middle school	8.046 (7.897 to 8.195)	3.470 (3.338 to 3.601)	2.020 (1.906 to 2.134)	−** 0.276 (**−** 0.324 to **−** 0.228)**	− 0.003 (− 0.098 to 0.093)	0.092 (− 0.049 to 0.232)
High school	16.604 (16.224 to 16.984)	10.237 (9.850 to 10.623)	7.170 (6.810 to 7.529)	−** 0.562 (**−** 0.691 to **−** 0.433)**	−** 0.414 (**−** 0.702 to **−** 0.126)**	0.004 (− 0.449 to 0.458)
BMI^b^						
Underweight	10.340 (10.110 to 10.570)	4.676 (4.452 to 4.901)	3.035 (2.806 to 3.264)	−** 0.335 (**−** 0.410 to **−** 0.259)**	−** 0.335 (**−** 0.498 to **−** 0.172)**	0.050 (− 0.228 to 0.328)
Normal	11.991 (11.761 to 12.221)	6.631 (6.393 to 6.868)	4.460 (4.240 to 4.680)	−** 0.350 (**−** 0.425 to **−** 0.274)**	−** 0.195 (**−** 0.369 to **−** 0.022)**	− 0.043 (− 0.317 to 0.230)
Overweight	11.868 (11.593 to 12.143)	7.661 (7.363 to 7.959)	5.053 (4.765 to 5.342)	−** 0.320 (**−** 0.413 to **−** 0.226)**	− 0.053 (− 0.271 to 0.165)	0.166 (− 0.205 to 0.538)
Economic status						
Low	16.364 (16.011 to 16.717)	9.905 (9.467 to 10.344)	7.205 (6.695 to 7.714)	−** 0.493 (**−** 0.625 to **−** 0.362)**	−** 0.425 (**−** 0.738 to **−** 0.112)**	0.266 (− 0.366 to 0.898)
Average	10.552 (10.348 to 10.756)	5.979 (5.759 to 6.200)	3.944 (3.742 to 4.146)	−** 0.295 (**−** 0.361 to **−** 0.228)**	− 0.075 (− 0.238 to 0.088)	0.053 (− 0.205 to 0.311)
High	9.759 (9.531 to 9.986)	5.810 (5.590 to 6.030)	3.987 (3.768 to 4.205)	−** 0.382 (**−** 0.461 to **−** 0.303)**	− 0.066 (− 0.222 to 0.090)	− 0.015 (− 0.288 to 0.258)
Academic performance						
Low	18.138 (17.825 to 18.451)	10.573 (10.225 to 10.921)	7.605 (7.267 to 7.944)	−** 0.723 (**−** 0.836 to **−** 0.610)**	− 0.119 (− 0.373 to 0.135)	− 0.199 (− 0.622 to 0.225)
Average	9.653 (9.427 to 9.879)	5.352 (5.112 to 5.592)	3.320 (3.101 to 3.540)	−** 0.332 (**−** 0.403 to **−** 0.262)**	−** 0.224 (**−** 0.400 to **−** 0.048)**	0.122 (− 0.151 to 0.395)
High	6.572 (6.401 to 6.744)	3.853 (3.679 to 4.027)	2.390 (2.222 to 2.557)	−** 0.266 (**−** 0.327 to **−** 0.204)**	− 0.066 (− 0.191 to 0.059)	**0.243 (0.033 to 0.453)**
Residence						
Rural	13.855 (13.207 to 14.504)	7.583 (6.647 to 8.519)	5.673 (4.807 to 6.539)	− 0.104 (− 0.312 to 0.103)	− 0.191 (− 0.812 to 0.430)	0.085 (− 1.081 to 1.252)
Suburban	11.717 (11.393 to 12.042)	6.657 (6.356 to 6.959)	4.618 (4.343 to 4.892)	−** 0.388 (**−** 0.495 to **−** 0.281)**	− 0.147 (− 0.368 to 0.075)	0.068 (− 0.281 to 0.417)
Urban	11.029 (10.764 to 11.295)	6.101 (5.798 to 6.404)	3.780 (3.531 to 4.029)	−** 0.324 (**−** 0.412 to **−** 0.235)**	− 0.187 (− 0.409 to 0.035)	− 0.056 (− 0.362 to 0.251)
Educational level of parents						
Middle school or lower	17.419 (16.803 to 18.034)	11.551 (10.142 to 12.961)	8.372 (6.394 to 10.351)	0.019 (− 0.213 to 0.250)	0.158 (− 0.873 to 1.189)	− 0.526 (− 2.811 to 1.759)
High school	13.585 (13.321 to 13.849)	8.650 (8.287 to 9.014)	5.608 (5.220 to 5.996)	−** 0.193 (**−** 0.281 to **−** 0.105)**	− 0.175 (− 0.448 to 0.098)	0.197 (− 0.295 to 0.690)
College or higher	9.268 (9.065 to 9.470)	5.424 (5.224 to 5.624)	3.297 (3.119 to 3.475)	−** 0.244 (**−** 0.313 to **−** 0.174)**	−** 0.170 (**−** 0.319 to **−** 0.021)**	0.001 (− 0.223 to 0.225)
Familial secondhand smoking						
No	8.566 (8.379 to 8.753)	5.271 (5.083 to 5.458)	3.979 (3.794 to 4.164)	−** 0.127 (**−** 0.196 to **−** 0.057)**	− 0.079 (− 0.217 to 0.060)	− 0.063 (− 0.297 to 0.171)
Yes	16.179 (15.873 to 16.485)	9.602 (9.265 to 9.940)	5.432 (5.130 to 5.735)	−** 0.625 (**−** 0.743 to **−** 0.508)**	−** 0.360 (**−** 0.597 to **−** 0.124)**	0.330 (− 0.046 to 0.705)
Stress conditions						
Low	8.953 (8.700 to 9.205)	5.527 (5.260 to 5.794)	3.507 (3.219 to 3.794)	−** 0.200 (**−** 0.282 to -0.118)**	− 0.070 (− 0.260 to 0.119)	− 0.101 (− 0.454 to 0.252)
Avg	10.017 (9.797 to 10.236)	5.764 (5.540 to 5.989)	3.497 (3.302 to 3.693)	−** 0.257 (**−** 0.329 to **−** 0.184)**	−** 0.233 (**−** 0.398 to **−** 0.067)**	0.005 (− 0.233 to 0.243)
High	13.930 (13.675 to 14.186)	7.768 (7.490 to 8.045)	5.683 (5.414 to 5.952)	−** 0.375 (**−** 0.461 to **−** 0.290)**	−** 0.208 (**−** 0.412 to **−** 0.005)**	− 0.116 (− 0.464 to 0.232)
Suicidal ideation within a year						
No	10.141 (9.945 to 10.337)	5.955 (5.750 to 6.161)	3.751 (3.573 to 3.930)	−** 0.272 (**−** 0.336 to **−** 0.207)**	−** 0.215 (**−** 0.366 to **−** 0.065)**	− 0.070 (− 0.292 to 0.153)
Yes	17.386 (17.046 to 17.726)	10.230 (9.807 to 10.653)	8.377 (7.892 to 8.863)	−** 0.242 (**−** 0.355 to **−** 0.128)**	0.049 (− 0.253 to 0.350)	0.013 (− 0.593 to 0.620)
Alcohol consumption within a month						
No	8.320 (8.120 to 8.520)	6.165 (5.904 to 6.426)	5.355 (5.037 to 5.673)	**0.367 (0.299 to 0.435)**	−** 0.219 (**−** 0.414 to **−** 0.024)**	− 0.220 (− 0.617 to 0.178)
Yes	12.949 (12.717 to 13.181)	6.571 (6.349 to 6.794)	4.034 (3.847 to 4.222)	−** 0.649 (**−** 0.725 to **−** 0.573)**	− 0.148 (− 0.309 to 0.013)	0.099 (-0.140 to 0.337)

CSP, current smoking prevalence; CI, confidence interval; KYRBS, Korea Youth Risk Behavior Web-Based Survey; SE, standard error.

Numbers in bold indicate a significant difference (*P* < 0.05).

^a^All βs are expressed by multiplying 100.

^b^BMI was divided into three groups according to the KDCA: underweight (< 18.5 kg/m^2^); normal (18.5 to 22.9 kg/m^2^); and overweight (≥ 23.0 kg/m^2^).

Table 3 presents the results of ITSA on CSP. The tobacco tax increase in 2015 was associated with an immediate reduction in CSP by − 3.076 [95% CI − 3.707 to − 2.445], with a pronounced immediate decrease among adolescents of lower economic status, lower academic performance, residing in rural areas, having parents with lower educational levels, and experiencing suicidal ideation within a year. The COVID-19 pandemic was associated with a small immediate decrease in CSP of − 1.833 [95% CI − 2.434 to − 1.232]. Considering the difference in trend associated with the two interruptions, CSP continued to decrease at a similar rate before and after the tobacco tax increase (β_diff_: 0.169 [95% CI 0.005–0.333]). Furthermore, while the overall difference in trend before and during the COVID-19 pandemic is insignificant (0.184 [95% CI − 0.092 to 0.461]), the difference in trend varied considerably depending on the characteristics of the adolescent. Particularly, adolescents with underweight BMI (0.385 [95% CI 0.063–0.707]), lower socioeconomic status (0.692 [95% CI − 0.013 to 1.397]), higher academic performance (0.309 [95% CI 0.065–0.553]), and secondhand smoking within the family (0.690 [95% CI 0.247–1.134]) showed significant increasing trend in CSP.

**Table 3:** Interrupted time series analysis of current smoking prevalence and tobacco tax increase and the COVID-19 pandemic from 2005 to 2022, of adolescents in South Korea by subgroups.

CSP	Sustained effect		Immediate effect	
	Tobacco tax increase	COVID-19 pandemic	Tobacco tax increase	COVID-19 pandemic
Overall	**0.169 (0.005 to 0.333)**	0.184 (− 0.092 to 0.461)	− **3.076 (**− **3.707 to **− **2.445)**	− **1.833 (**− **2.434 to **− **1.232)**
Sex				
Male	− **0.464 (**− **0.702 to **− **0.225)**	**0.619 (0.201 to 1.037)**	− **4.089 (**− **4.993 to **− **3.186)**	− **2.658 (**− **3.573 to **− **1.743)**
Female	**0.852 (0.721 to 0.984)**	− **0.283 (**− **0.513 to **− **0.054)**	− **1.926 (**− **2.378 to **− **1.474)**	− **0.945 (**− **1.472 to **− **0.418)**
Grade				
Middle school	**0.273 (0.167 to 0.380)**	0.094 (− 0.075 to 0.264)	− **3.311 (**− **3.708 to **− **2.914)**	− **1.628 (**− **2.007 to **− **1.248)**
High school	0.148 (− 0.167 to 0.463)	0.418 (− 0.119 to 0.955)	− **2.719 (**− **3.922 to **− **1.516)**	− **2.207 (**− **3.367 to **− **1.047)**
BMI^a^				
Underweight	0.000 (− 0.180 to 0.179)	**0.385 (0.063 to 0.707)**	− **3.186 (**− **3.864 to **− **2.509)**	− **1.021 (**− **1.729 to **− **0.313)**
Normal	0.154 (− 0.035 to 0.344)	0.152 (− 0.172 to 0.476)	− **3.237 (**− **3.955 to **− **2.518)**	− **1.666 (**− **2.369 to **− **0.962)**
Overweight	**0.266 (0.029 to 0.503)**	0.220 (− 0.211 to 0.651)	− **2.650 (**− **3.538 to **− **1.763)**	− **2.830 (**− **3.763 to **− **1.897)**
Economic status				
Low	0.068 (− 0.272 to 0.407)	0.692 (− 0.013 to 1.397)	− **3.165 (**− **4.402 to **− **1.929)**	− **2.253 (**− **3.752 to **− **0.754)**
Average	**0.219 (0.044 to 0.395)**	0.128 (− 0.176 to 0.433)	− **2.952 (**− **3.611 to **− **2.292)**	− **1.982 (**− **2.637 to **− **1.326)**
High	**0.316 (0.141 to 0.491)**	0.051 (− 0.263 to 0.366)	− **2.192 (**− **2.831 to **− **1.553)**	− **1.658 (**− **2.378 to **− **0.937)**
Academic performance				
Low	**0.604 (0.326 to 0.882)**	− 0.080 (− 0.573 to 0.414)	− **4.207 (**− **5.220 to **− **3.194)**	− **2.320 (**− **3.406 to **− **1.234)**
Average	0.108 (− 0.081 to 0.297)	**0.346 (0.022 to 0.671)**	− **1.982 (**− **2.712 to **− **1.253)**	− **1.810 (**− **2.516 to **− **1.104)**
High	**0.199 (0.060 to 0.339)**	**0.309 (0.065 to 0.553)**	− **1.386 (**− **1.893 to **− **0.880)**	− **1.813 (**− **2.347 to **− **1.279)**
Residence				
Rural	− 0.087 (− 0.741 to 0.568)	0.276 (− 1.045 to 1.597)	− **5.247 (**− **7.723 to **− **2.772)**	− 1.668 (− 4.308 to 0.971)
Suburban	**0.241 (**− **0.005 to 0.487)**	0.215 (− 0.199 to 0.628)	− **2.951 (**− **3.886 to **− **2.015)**	− **1.868 (**− **2.771 to **− **0.965)**
Urban	0.137 (− 0.102 to 0.375)	0.131 (− 0.247 to 0.510)	− **2.903 (**− **3.828 to **− **1.979)**	− **1.814 (**− **2.646 to **− **0.982)**
Educational level of parents				
Middle school or lower	0.139 (− 0.917 to 1.196)	− 0.684 (− 3.190 to 1.821)	− **6.368 (**− **9.671 to **− **3.065)**	− 2.527 (− 8.429 to 3.376)
High school	0.018 (− 0.269 to 0.304)	0.372 (− 0.190 to 0.935)	− **3.553 (**− **4.564 to **− **2.541)**	− **3.009 (**− **4.206 to **− **1.812)**
College or higher	0.074 (− 0.090 to 0.237)	0.171 (− 0.098 to 0.439)	− **2.340 (**− **2.939 to **− **1.741)**	− **1.755 (**− **2.355 to **− **1.156)**
Familial secondhand smoking				
No	0.048 (− 0.107 to 0.203)	0.016 (− 0.256 to 0.288)	− **2.576 (**− **3.157 to **− **1.995)**	− **0.999 (**− **1.600 to **− **0.398)**
Yes	**0.265 (0.001 to 0.529)**	**0.690 (0.247 to 1.134)**	− **2.816 (**− **3.785 to **− **1.847)**	− **4.055 (**− **4.984 to **− **3.126)**
Stress conditions				
Low	0.130 (− 0.077 to 0.336)	− 0.030 (− 0.431 to 0.370)	− **2.375 (**− **3.132 to **− **1.617)**	− **1.676 (**− **2.571 to **− **0.781)**
Avg	0.024 (− 0.157 to 0.205)	0.238 (− 0.052 to 0.528)	− **2.459 (**− **3.135 to **− **1.783)**	− **1.778 (**− **2.410 to **− **1.145)**
High	0.167 (− 0.054 to 0.388)	0.093 (− 0.310 to 0.496)	− **3.809 (**− **4.654 to **− **2.964)**	− **1.420 (**− **2.293 to **− **0.547)**
Suicidal ideation within a year				
No	0.056 (− 0.107 to 0.220)	0.146 (− 0.123 to 0.414)	− **2.376 (**− **3.000 to **− **1.751)**	− **1.610 (**− **2.202 to **− **1.018)**
Yes	0.290 (− 0.032 to 0.612)	− 0.035 (− 0.712 to 0.642)	− **6.122 (**− **7.301 to **− **4.942)**	− **1.980 (**− **3.459 to **− **0.500)**
Alcohol consumption within a month				
No	− **0.586 (**− **0.793 to **− **0.379)**	− 0.001 (− 0.444 to 0.442)	− **3.208 (**− **3.960 to **− **2.455)**	0.079 (− 0.928 to 1.087)
Yes	**0.501 (0.323 to 0.679)**	0.246 (− 0.041 to 0.534)	− **3.082 (**− **3.765 to **− **2.400)**	− **2.421 (**− **3.040 to **− **1.803)**

CSP, current smoking prevalence; CI, confidence interval; KYRBS, Korea Youth Risk Behavior Web-Based Survey.

Numbers in bold indicate a significant difference (*P* < 0.05).

^a^BMI was divided into three groups according to the KDCA: underweight (< 18.5 kg/m^2^); normal (18.5–22.9 kg/m^2^); overweight (≥ 23.0 kg/m^2^).

Sustained effect of tobacco tax increase, sustained effect of COVID-19 pandemic, immediate effect of tobacco tax increase, immediate effect of COVID-19 pandemic each indicates coefficient α_2_, α_3_, α_4_, and α_5_ calculated by the interrupted time series analysis.

Table S2 presents the results of ITSA on DSP. The tobacco tax increase was associated with an immediate reduction in DSP by − 4.112 [95% CI − 6.488 to − 1.735]. The COVID-19 pandemic was associated with a strong immediate increase in DSP of 9.345 [95% CI 5.285–13.406], with particularly strong increase among adolescents with lower socioeconomic status (8.308 [95% CI 0.133–16.482]), lower academic performance (9.122 [95% CI 4.011–14.232]), urban residency (13.546 [95% CI 7.427–19.664]), under parents with lower educational level (31.544 [95% CI 3.030–60.059]), exposed to secondhand smoking within the family (15.168 [95% CI 8.417–21.919]), higher stress condition (12.396 [95% CI 6.863–17.930]) and experienced suicidal ideation within a year (11.944 [95% CI 3.960–19.927]). Considering the difference in DSP trend associated with the tobacco tax increase, the reduction rate of DSP even more decreased (β_diff_: − 1.893 [95% CI − 2.587 to − 1.198]), with pronounced effect among adolescents with underweight BMI, lower educational level of parents, experiencing secondhand smoking within the family, and having suicidal ideation within a year.

## Discussion

Conducted on a nationwide representative scale, this study analyzed the long-term trends (2005–2022) of CSP and DSP, influenced by the increase in tobacco taxes and the COVID-19 pandemic, using ITSA. The study’s principal findings are outlined as follows. First, overall CSP decreased while DSP increased over the 3 periods (pre-tax-increase period, post-tax-increase period,and pandemic). Notably, CSP exhibited a decreasing trend during the pre-pandemic, which disappeared during the pandemic. Conversely, DSP, previously characterized by an increasing trend before the pandemic, changed to a decreasing trend during the pandemic. Second, we found an intriguing association surfaced between the COVID-19 pandemic and a substantial short-term increase in DSP. This was particularly prominent among adolescents with lower economic status, lower educational level of parents, familial secondhand smoke, poor mental health conditions, and lower academic performance. Finally, the COVID-19 pandemic was associated with a long-term increase in CSP among adolescents of lower economic status, with exposure to familial secondhand smoke, and higher academic performance. Taken together, we suggest that the pandemic significantly aggravates the smoking prevalence of adolescents of lower economic status, and exposed to familial secondhand smoke, both in short and long terms.

This study demonstrates that while the overall percentage of current smokers among adolescents is on the decline, there is an upward trajectory in the proportion of daily smokers among adolescents who do smoke. This suggests that despite the diminishing numbers of adolescent smokers, there is an increasing likelihood for those who are currently smoking to develop a tobacco dependence.

We observed that a rise in tobacco taxes was associated with an immediate reduction in both DSP and CSP. These outcomes can be attributed to the notion that a tobacco tax increase establishes economic deterrents to smoking, thereby contributing to the overall reduction in smoking prevalence among adolescents^14,28^. Moreover, the advent of the COVID-19 pandemic resulted in an immediate decrease in CSP, potentially due to strict restrictions on outdoor activities and the necessity for social distancing^15,29^.

Significantly, the COVID-19 pandemic was associated with an immediate increase in DSP, and a sustained increase in CSP, particularly prominent among adolescents from lower economic status. Furthermore, regarding the immediate increase in DSP, adolescents with poor mental health and lower academic performance experienced the most notable immediate increase. Conversely, concerning the sustained increase in CSP associated with the COVID-19 pandemic, adolescents with high academic performance notably shifted from a previously declining trend observed across all academic performance levels before the pandemic to a significant increasing trend. Stress and depression resulting from the prolonged duration of the COVID-19 pandemic may have led even adolescents who originally had good mental health and high academic performance to resort to smoking as a means of stress relief^30^. The link between these factors and adolescent smoking behavior may be attributed to stress and anxiety, known triggers for smoking, heightened by the pandemic’s circumstances^16,19^. Smoking is occasionally employed as a coping mechanism for stress relief, and the pandemic-induced social isolation and anxiety might have contributed to the increasing trend of CSP to adolescents with high academic performance^17^.

The influence of the tobacco tax increase and the COVID-19 pandemic on adolescent CSP have been demonstrated in several studies^14,18^. Our study showed consistent results with most previous studies; USA^31^ (n = 10,981), England (n = 274,890)^32^, China^33^ (n = 419,875), Sweden^34^ (n = 1818), and China (n = 31,273)^35^. However, most previous studies did not focus on the prevalence of daily smokers among those who currently smoke. Furthermore, the significant impact of the tobacco tax increase was not consistently factored into most COVID-19 pandemic-related studies^14^. With this background, we undertook an approach by devising and implementing an interrupted time-series model that accounted for the influence of both the tobacco tax increase and the COVID-19 pandemic as interventions. Moreover, our study performed numerous stratification analyses to enhance the robustness of our key findings and to discern which specific subgroup of adolescents was particularly vulnerable during the pandemic.

This study has several limitations. First, adolescents were stratified according to self-reported data, such as economic level and academic performance, introducing the possibility of social desirability bias and recall bias. Furthermore, the increase in the proportion of individuals from low economic status over the study period raised the possibility of selection bias. While we aimed to account for these factors through weighting techniques to adjust for sampling probabilities and nonresponse rates across demographic groups, residual selection bias may persist. In the context of the STROBE (Strengthening the Reporting of Observational Studies in Epidemiology) guideline^36^, it is crucial to recognize that reliance on self-reported data may impact the external validity of our findings. Second, the analysis solely focused on data from Korean adolescents, and caution should be exercised when generalizing the trends to other countries. Cultural and regional differences may influence smoking behavior differently in diverse populations. Thirdly, the use of ITSA in our study introduces the potential for unmeasured confounding variables, such as other tobacco-related factors or negative cultural stigma surrounding smoking^37,38^. Although we attempted to control for potential confounders in our analysis, including socioeconomic variables and temporal trends, the presence of unmeasured factors could still influence the observed outcomes. In accordance with the STROBE guideline, further research may be needed to explore and address these potential confounders comprehensively. Next, our data were collected on an annual basis, limiting our ability to adequately adjust for seasonality in our analysis. Future studies should consider more frequent data collection methods to better account for seasonal fluctuations in adolescent smoking behavior, aligning with the STROBE guideline's recommendations. Finally, it is important to acknowledge that conducting multiple tests increases the possibility of falsely rejecting null hypotheses. However, given that different criteria used for dividing the overall sample did not affect each other, and consistent weighting was applied throughout our analysis, we believe that the impact of conducting multiple tests on our results seems minimal. Nonetheless, it's worth noting that certain subgroups with smaller sample sizes may pose an additional limitation to our study.

However, the current study has several strengths. First, our study has a large sample size (n = 1,192,578) and shows representative data of the Korean population. Second, a comprehensive investigation of stratified subgroups was analyzed, thus, effectively showing the impact of tobacco tax increase and the COVID-19 pandemic under different characteristics of adolescents. Third, the interrupted time-series model was expanded to analyze two unique events, by adjusting for the effects of one event while analyzing the other, this approach allows for a more accurate assessment of the individual impacts of each event. Specifically, the tobacco tax increase was used as a factor for adjusting the potential confounding factors when assessing the impact of the COVID-19 pandemic on smoking behavior, and vice versa. Finally, by using an interrupted time-series model we were able to study the short-term and long-term association separately.

The findings of this study have substantial implications for policies concerning smoking prevention and addiction management among adolescents. Although our current initiatives have effectively reduced CSP, it is alarming that DSP showed an increasing trend during the COVID-19 pandemic. Prior research suggests that daily smoking is associated with nicotine addiction, indicating that adolescents who smoke daily may have to increased risk of developing nicotine addiction^39^. Therefore, by associating with the analyzed DSP trends, increase in tobacco tax potentially lead to an decrease in the progression towards nicotine addiction among adolescents. This highlights the need to expand our strategies beyond preventing adolescents from initiating smoking and to develop policies or education programs for smoking cessation. Additionally, programs such as peer support and mentorship, school-based cessation education, and applications targeted for adolescent smokers will be helpful, especially when they target those already engaged in daily smoking^20^.

Tobacco tax increase policies have proven highly effective in reducing CSP, as our study reaffirms^19,40^. In addition, we observed a significant reduction in the prevalence of daily smokers among current smokers following tobacco tax increases. This highlights the potential of tobacco tax policies not only in curbing the initiation of smoking among adolescents but also in impeding the progression towards nicotine addiction. Therefore, policymakers must consider the strategic implementation of tobacco tax increases, focusing on research efforts that maximize immediate effects, as our findings suggest that the immediate impact of tax increases is notably stronger, resulting in a decrease in both CSP and DSP.

The COVID-19 pandemic has left a significant impact on the smoking behavior of adolescents. Our research uncovers an immediate surge in DSP and a persistent increase in CSP during this period. This trend is particularly pronounced among adolescents hailing from lower economic backgrounds and those exposed to familial secondhand smoke. Additionally, it's worth noting that adolescents with higher academic performance tend to exhibit a stronger association with long-term association, while their counterparts with lower academic performance are more inclined to experience short-term consequences. To effectively address the pandemic's adverse effects on smoking behavior, policymakers are encouraged to implement a comprehensive approach that considers the pandemic's long-term consequences, socioeconomic factors, and family environment, and particularly focuses on tailored interventions for vulnerable populations such as adolescents from lower economic status or those exposed to secondhand smoke within their families: Support for low-income families: Implement targeted support and programs for adolescents from low-income families to significantly reduce both CSP and DSP^41,42^. These initiatives should encompass not only smoking prevention but also address socioeconomic disparities; Comprehensive family-oriented approach: Prioritize a family-oriented approach to smoking prevention, including education and support for adolescents and their families. This approach aims to create a smoke-free home environment and provide necessary resources; Stress and anxiety mitigation: Recognize the influence of pandemic-induced stress and anxiety on smoking behaviors. Implement policies that alleviate these psychological burdens by strengthening emotional support systems and offering stress management programs^43^. These measures can play a crucial role in reducing both CSP and DSP.

## Conclusion

This study utilized ITSA to observe both the long-term and short-term association of the tobacco tax increase and the COVID-19 pandemic on adolescent smoking behavior from 2005 to 2022. While an overall decline in CSP was evident, the advent of the COVID-19 pandemic changed the decreasing trend to an increasing trend. Similarly, in DSP, the pandemic engendered a shift from the pre-pandemic rising trend to a diminishing trend. The tobacco tax increase reduced smoking prevalence among adolescents in both the short and long terms, however, the impact in the short-term was more pronounced. On the other hand, the COVID-19 pandemic was linked to an immediate increase in DSP, and a sustained increase in CSP, particularly pronounced among specific adolescent subgroups. An immediate increase in DSP leads to a rise in daily smoking. This increase raises public concerns about adolescent health, as daily smoking is more strongly associated with nicotine addiction or negative health issues. Adolescents of low economic status and exposed to secondhand smoke within their families experienced the strongest impact of the COVID-19 pandemic. Therefore, implementing policies aimed at economically supporting adolescents from low-income families and to minimize second-hand smoke exposure would be beneficial. In the immediate term, adolescents of low economic status with poor mental health and low academic performance showed a high increase in DSP. Conversely, in the long-term, adolescents with high academic performance, economic status, and exposed to secondhand smoking within the family demonstrated an increasing trend of CSP.

### Supplementary Information

Supplementary Information.

## Data Availability

Data are available on reasonable request. Study protocol, statistical code: available from DKY (email: yonkkang@gmail.com). Data set: available from the Korean Disease Control and Prevention Agency (KDCA) through a data use agreement.
