# Supplementary material for: National prevalence of smoking among adolescents at tobacco tax increase and COVID-19 pandemic in South Korea, 2005–2022
Source: Sci Rep. 2024 Apr 3;14:7823. doi: 10.1038/s41598-024-58446-4 (PMC10991517; doi:10.1038/s41598-024-58446-4)
Supplement: Supplementary file 1 — Supplementary Information. [file 41598_2024_58446_MOESM1_ESM.docx]

| **Supplementary Material** |
| --- |

**National prevalence of smoking among adolescents at tobacco tax increase and COVID–19 pandemic in South Korea, 2005–2022**

Seohyun Hong,^1∥^ Selin Woo,^2,3∥^ Seokjun Kim, ^1^ Jaeyu Park,^2,3^ Myeongcheol Lee,^2,3^ Sunyoung Kim,^4^ Ai Koyanagi,^5^ Lee Smith,^6^ Min Seo Kim,^7^ [Guillermo F. López Sánchez](https://streaklinks.com/BfMTWjzVFXnjyxjmNQoEXiV-/https%3A%2F%2Fpubmed.ncbi.nlm.nih.gov%2F%3Fsort%3Ddate%26size%3D200%26term%3DL%25C3%25B3pez%2BS%25C3%25A1nchez%2BGF%26cauthor_id%3D36808652?email=yonkkang%40gmail.com),^8^ Elena Dragioti,^9,10^ Masoud Rahmati,^11,12^ Guillaume Fond,^13^ Laurent Boyer,^13^ Jiyeon Oh,^1*^ Hojae Lee,^2,3*^ Dong Keon Yon^1,2,3,14*^

∥ These authors contributed equally.

^*^ These authors contributed equally.

**Lead Contact**

**Dong Keon Yon, MD, PhD, FACAAI, FAAAAI**

Department of Pediatrics, Kyung Hee University College of Medicine, 23 Kyungheedae-ro, Dongdaemun-gu, Seoul 02447, South Korea.

Tel: +82-2-6935-2476

Fax: +82-504-478-0201

Email: [yonkkang@gmail.com](mailto:yonkkang@gmail.com)

**Figure S1.** Annual-level overall DSP of adolescents in South Korea between 2005 and 2022, weighted %, in the KYRBS. Data are as observed (measured, black line; trend, blue line) from an interrupted time-series analysis. Dashed red line shows predicted prevalence if the tobacco tax increase and the COVID-19 pandemic didn’t occur.


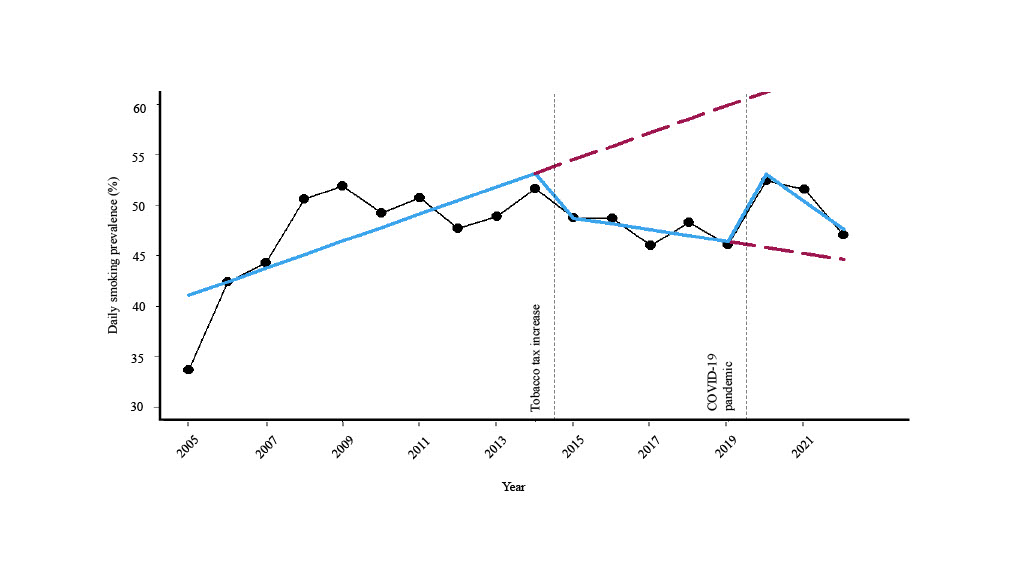


**Table S1.** The trend of the daily smoking prevalence of adolescents, β-coefficients before and after tobacco tax increase and the COVID-19 pandemic, weighted % (95% CI), in the KYRBS.

| **DSP** | Prevalence, weighted % (95% CI) | | | Trend analysis, β (95% CI) ^a^ | | |
| --- | --- | --- | --- | --- | --- | --- |
|  | Pre-tax increase-period  (2005-2014) | Post-tax increase-period  (2015-2019) | Pre-tax increase-period  (2005-2014) | Post-tax increase-period  (2015-2019) | Pre-tax increase-period  (2005-2014) | Post-tax increase-period  (2015-2019) |
| Overall | 47.251 (46.661 to 47.841) | 47.667 (46.710 to 48.624) | 50.372 (48.968 to 51.777) | **1.313 (1.107 to 1.518)** | -0.580 (-1.244 to 0.083) | **-2.697 (-4.463 to -0.931)** |
| Sex | | | | | | |
| Male | 50.554 (49.887 to 51.220) | 50.525 (49.491 to 51.559) | 53.750 (52.074 to 55.426) | **0.990 (0.753 to 1.228)** | 0.188 (-0.530 to 0.905) | -1.919 (-3.984 to 0.146) |
| Female | 38.670 (37.654 to 39.687) | 37.645 (35.851 to 39.439) | 41.988 (39.481 to 44.495) | **1.487 (1.154 to 1.819)** | **-1.832 (-3.124 to -0.540)** | **-4.774 (-7.896 to -1.653)** |
| Grade | | | | | | |
| Middle school | 30.788 (30.113 to 31.463) | 30.901 (29.524 to 32.277) | 34.911 (32.442 to 37.380) | **1.404 (1.162 to 1.646)** | 0.004 (-0.938 to 0.946) | -2.271 (-5.298 to 0.757) |
| High school | 58.878 (58.192 to 59.564) | 54.792 (53.697 to 55.887) | 55.746 (54.175 to 57.316) | **0.907 (0.664 to 1.150)** | -0.639 (-1.413 to 0.136) | **-2.462 (-4.432 to -0.492)** |
| BMI^b^ | | | | | | |
| Underweight | 45.142 (44.165 to 46.120) | 45.665 (43.582 to 47.748) | 47.829 (44.289 to 51.370) | **1.682 (1.334 to 2.030)** | -1.070 (-2.520 to 0.380) | -4.140 (-8.469 to 0.188) |
| Normal | 48.233 (47.510 to 48.955) | 47.734 (46.555 to 48.914) | 50.937 (48.957 to 52.918) | **1.284 (1.028 to 1.540)** | -0.791 (-1.614 to 0.032) | -1.928 (-4.430 to 0.573) |
| Overweight | 47.029 (45.997 to 48.061) | 48.572 (47.119 to 50.025) | 50.712 (48.503 to 52.920) | **0.972 (0.620 to 1.324)** | -0.094 (-1.091 to 0.903) | **-3.013 (-5.755 to -0.272)** |
| Economic status | | | | | | |
| Low | 51.538 (50.631 to 52.445) | 50.966 (49.043 to 52.890) | 51.707 (48.804 to 54.610) | **1.083 (0.737 to 1.430)** | -0.354 (-1.645 to 0.937) | -3.433 (-7.058 to 0.192) |
| Average | 45.445 (44.689 to 46.202) | 46.056 (44.778 to 47.333) | 49.692 (47.679 to 51.705) | **1.465 (1.209 to 1.721)** | **-0.932 (-1.833 to -0.031)** | **-2.683 (-5.215 to -0.151)** |
| High | 45.508 (44.523 to 46.494) | 47.611 (46.207 to 49.014) | 50.496 (48.131 to 52.861) | **1.068 (0.714 to 1.423)** | -0.043 (-1.016 to 0.929) | -2.338 (-5.257 to 0.582) |
| Academic performance | | | | | | |
| Low | 51.607 (50.908 to 52.306) | 51.478 (50.236 to 52.720) | 54.696 (52.916 to 56.476) | **0.833 (0.569 to 1.097)** | -0.544 (-1.396 to 0.307) | **-2.412 (-4.614 to -0.210)** |
| Average | 42.311 (41.346 to 43.277) | 43.618 (41.932 to 45.304) | 45.575 (42.873 to 48.277) | **1.704 (1.388 to 2.020)** | -1.049 (-2.206 to 0.107) | **-3.408 (-6.805 to -0.011)** |
| High | 41.730 (40.663 to 42.797) | 43.086 (41.366 to 44.805) | 44.228 (41.123 to 47.332) | **1.127 (0.727 to 1.528)** | -0.051 (-1.221 to 1.120) | -1.159 (-4.882 to 2.564) |
| Residence | | | | | | |
| Rural | 48.036 (46.319 to 49.754) | 49.190 (45.482 to 52.898) | 51.518 (45.825 to 57.212) | **1.556 (0.939 to 2.172)** | -1.335 (-4.101 to 1.432) | -1.175 (-8.641 to 6.291) |
| Suburban | 46.693 (45.733 to 47.653) | 47.460 (46.138 to 48.783) | 50.220 (48.243 to 52.197) | **1.214 (0.882 to 1.547)** | -0.520 (-1.448 to 0.409) | -1.509 (-4.011 to 0.993) |
| Urban | 47.691 (46.897 to 48.486) | 47.672 (46.166 to 49.178) | 50.374 (48.246 to 52.502) | **1.397 (1.122 to 1.672)** | -0.531 (-1.545 to 0.482) | **-4.893 (-7.473 to -2.313)** |
| Educational level of parents | | | | | | |
| Middle school or lower | 52.294 (50.404 to 54.185) | 50.989 (44.911 to 57.068) | 66.632 (56.281 to 76.984) | **1.721 (1.010 to 2.432)** | -3.803 (-8.247 to 0.641) | -3.349 (-15.285 to 8.587) |
| High school | 49.669 (48.900 to 50.437) | 50.309 (48.713 to 51.906) | 50.137 (47.049 to 53.224) | **1.475 (1.209 to 1.741)** | **-1.187 (-2.364 to -0.010)** | -2.491 (-6.374 to 1.393) |
| College or higher | 44.684 (43.887 to 45.481) | 45.286 (44.072 to 46.500) | 45.427 (43.139 to 47.715) | **1.507 (1.222 to 1.792)** | **-0.895 (-1.743 to -0.046)** | **-3.131 (-5.946 to -0.315)** |
| Familial secondhand smoking | | | | | | |
| No | 48.793 (48.022 to 49.563) | 48.765 (47.634 to 49.897) | 50.654 (48.959 to 52.349) | **0.654 (0.343 to 0.965)** | -0.335 (-1.107 to 0.436) | -1.867 (-3.937 to 0.203) |
| Yes | 48.235 (47.490 to 48.981) | 46.096 (44.757 to 47.436) | 49.712 (47.341 to 52.084) | **0.847 (0.552 to 1.142)** | **-0.981 (-1.936 to -0.025)** | **-4.678 (-7.648 to -1.708)** |
| Stress conditions | | | | | | |
| Low | 47.243 (45.943 to 48.542) | 48.582 (46.633 to 50.531) | 55.758 (52.373 to 59.142) | **1.522 (1.056 to 1.987)** | 0.284 (-1.058 to 1.627) | -1.270 (-5.451 to 2.911) |
| Avg | 45.800 (44.984 to 46.617) | 46.091 (44.717 to 47.465) | 47.501 (45.216 to 49.787) | **1.296 (1.011 to 1.580)** | 0.211 (-0.780 to 1.202) | -2.102 (-4.923 to 0.720) |
| High | 48.250 (47.539 to 48.962) | 48.655 (47.312 to 49.998) | 50.718 (48.797 to 52.639) | **1.314 (1.061 to 1.567)** | **-1.603 (-2.518 to -0.688)** | **-3.410 (-5.744 to -1.076)** |
| Suicidal ideation within a year | | | | | | |
| No | 48.081 (47.428 to 48.734) | 48.538 (47.505 to 49.570) | 51.762 (50.128 to 53.396) | **1.224 (0.998 to 1.449)** | -0.191 (-0.908 to 0.526) | **-2.410 (-4.451 to -0.370)** |
| Yes | 45.208 (44.312 to 46.105) | 43.984 (42.161 to 45.806) | 45.979 (43.306 to 48.652) | **1.447 (1.121 to 1.773)** | **-1.942 (-3.174 to -0.710)** | -2.876 (-6.226 to 0.474) |
| Alcohol consumption within a month | | | | | | |
| No | 38.881 (37.916 to 39.847) | 39.780 (38.092 to 41.468) | 45.503 (43.066 to 47.940) | **1.849 (1.492 to 2.206)** | -0.313 (-1.495 to 0.870) | -1.921 (-5.002 to 1.161) |
| Yes | 49.633 (48.994 to 50.271) | 50.052 (48.993 to 51.110) | 52.199 (50.598 to 53.800) | **1.361 (1.140 to 1.583)** | -0.662 (-1.392 to 0.068) | **-3.181 (-5.156 to -1.205)** |

Abbreviations: DSP, daily smoking prevalence; CI, confidence interval; KYRBS, Korea Youth Risk Behavior Web-Based Survey; SE, standard error

Numbers in bold indicate a significant difference (*P* < 0.05).

^a^ All βs are expressed by multiplying 100.

^b^ BMI was divided into three groups according to the KDCA: underweight (<18.5 kg/m^2^); normal (18.5 to 22.9 kg/m^2^); overweight (≥23.0 kg/m^2^).

**Table S2.** Interrupted time series analysis of annual daily smoking prevalence and tobacco tax increase and the COVID-19 pandemic from 2005 to 2022, of adolescents in South Korea by subgroups

| **DSP** | Sustained effect | | Immediate effect | | |
| --- | --- | --- | --- | --- | --- |
|  | Tobacco tax increase | COVID-19 pandemic | Tobacco tax increase | COVID-19 pandemic | |
| Overall | **-1.893 (-2.587 to -1.198)** | **-2.117 (-4.002 to -0.232)** | **-4.112 (-6.488 to -1.735)** | **9.345 (5.285 to 13.406)** | |
| Sex | | | | | |
| Male | **-0.803 (-1.558 to -0.047)** | -2.107 (-4.291 to 0.078) | **-5.041 (-7.573 to -2.508)** | **6.644 (1.919 to 11.370)** | |
| Female | **-3.319 (-4.652 to -1.985)** | -2.943 (-6.319 to 0.434) | -3.057 (-7.849 to 1.735) | **17.389 (10.202 to 24.576)** | |
| Grade | | | | | |
| Middle school | **-1.400 (-2.372 to -0.428)** | -2.275 (-5.443 to 0.894) | **-6.685 (-9.904 to -3.467)** | **8.616 (1.528 to 15.705)** | |
| High school | **-1.546 (-2.357 to -0.734)** | -1.823 (-3.939 to 0.292) | **-6.468 (-9.211 to -3.726)** | **7.231 (2.676 to 11.787)** | |
| BMI ^a^ | | | | | |
| Underweight | **-2.752 (-4.243 to -1.260)** | -3.070 (-7.632 to 1.492) | -4.674 (-9.478 to 0.130) | **13.084 (3.175 to 22.994)** | |
| Normal | **-2.074 (-2.936 to -1.213)** | -1.137 (-3.769 to 1.494) | **-4.319 (-7.222 to -1.416)** | **8.759 (3.078 to 14.440)** | |
| Overweight | **-1.066 (-2.123 to -0.009)** | **-2.920 (-5.835 to -0.004)** | -2.639 (-6.424 to 1.145) | **8.357 (2.174 to 14.540)** | |
| Economic status | | | | | |
| Low | **-1.437 (-2.773 to -0.101)** | -3.079 (-6.924 to 0.767) | **-4.523 (-8.866 to -0.181)** | **8.308 (0.133 to 16.482)** | |
| Average | **-2.397 (-3.333 to -1.461)** | -1.751 (-4.437 to 0.935) | **-4.041 (-7.178 to -0.904)** | **10.972 (5.253 to 16.691)** | |
| High | **-1.112 (-2.147 to -0.077)** | -2.294 (-5.369 to 0.781) | -2.445 (-5.994 to 1.105) | **7.693 (1.092 to 14.295)** | |
| Academic performance | | | | | |
| Low | **-1.378 (-2.269 to -0.486)** | -1.867 (-4.227 to 0.492) | -2.297 (-5.245 to 0.651) | **9.122 (4.011 to 14.232)** | |
| Average | **-2.753 (-3.952 to -1.555)** | -2.359 (-5.944 to 1.227) | **-4.782 (-8.913 to -0.651)** | **11.100 (3.342 to 18.858)** | |
| High | -1.178 (-2.415 to 0.059) | -1.109 (-5.009 to 2.791) | -3.676 (-8.072 to 0.720) | 3.660 (-4.752 to 12.073) | |
| Residence | | | | | |
| Rural | **-2.891 (-5.724 to -0.057)** | 0.160 (-7.798 to 8.117) | -2.364 (-11.443 to 6.715) | 7.593 (-7.723 to 22.909) | |
| Suburban | **-1.734 (-2.720 to -0.748)** | -0.989 (-3.656 to 1.678) | **-3.333 (-6.712 to 0.045)** | **6.904 (1.150 to 12.658)** | |
| Urban | **-1.928 (-2.978 to -0.879)** | **-4.362 (-7.131 to -1.592)** | **-5.250 (-8.889 to -1.611)** | **13.546 (7.427 to 19.664)** | |
| Educational level of parents | | | | | |
| Middle school or lower | **-5.524 (-10.023 to -1.025)** | 0.454 (-12.274 to 13.182) | -1.178 (-14.253 to 11.898) | **31.544 (3.030 to 60.059)** | |
| High school | **-2.662 (-3.868 to -1.455)** | -1.304 (-5.359 to 2.751) | -3.510 (-7.274 to 0.254) | 7.583 (-1.026 to 16.192) | |
| College or higher | **-2.402 (-3.296 to -1.507)** | -2.236 (-5.175 to 0.703) | **-3.607 (-6.614 to -0.599)** | **8.502 (1.812 to 15.191)** | |
| Familial secondhand smoking | | | | | |
| No | **-0.990 (-1.822 to -0.158)** | -1.532 (-3.740 to 0.676) | -1.666 (-4.500 to 1.169) | **6.324 (1.562 to 11.085)** |  |
| Yes | **-1.828 (-2.828 to -0.828)** | **-3.697 (-6.815 to -0.579)** | **-3.282 (-6.572 to 0.007)** | **15.168 (8.417 to 21.919)** | |
| Stress conditions | | | | | |
| Low | -1.238 (-2.658 to 0.183) | -1.554 (-5.943 to 2.834) | **-6.204 (-10.948 to -1.460)** | **8.961 (0.070 to 17.852)** | |
| Avg | **-1.084 (-2.115 to -0.054)** | -2.313 (-5.302 to 0.676) | **-6.258 (-9.631 to -2.884)** | 5.092 (-1.367 to 11.551) | |
| High | **-2.916 (-3.865 to -1.967)** | -1.807 (-4.312 to 0.697) | -1.305 (-4.537 to 1.927) | **12.396 (6.863 to 17.930)** | |
| Suicidal ideation within a year | | | | | |
| No | **-1.415 (-2.166 to -0.664)** | **-2.219 (-4.381 to -0.058)** | **-4.627 (-7.204 to -2.050)** | **8.389 (3.834 to 12.945)** | |
| Yes | **-3.389 (-4.663 to -2.115)** | -0.934 (-4.501 to 2.633) | -2.671 (-7.107 to 1.764) | **11.944 (3.960 to 19.927)** | |
| Alcohol consumption within a month | | | | | |
| No | **-2.161 (-3.396 to -0.926)** | -1.608 (-4.907 to 1.691) | **-6.124 (-10.234 to -2.014)** | **10.130 (3.094 to 17.167)** | |
| Yes | **-2.023 (-2.786 to -1.260)** | **-2.519 (-4.624 to -0.414)** | **-4.272 (-6.894 to -1.650)** | **9.993 (5.485 to 14.501)** | |

Abbreviations: DSP, daily smoking prevalence; CI, confidence interval; KYRBS, Korea Youth Risk Behavior Web-Based Survey

Numbers in bold indicate a significant difference (*P* < 0.05).

^a^ BMI was divided into three groups according to the KDCA: underweight (<18.5 kg/m^2^); normal (18.5 to 22.9 kg/m^2^); overweight (≥23.0 kg/m^2^).

Sustained effect of tobacco tax increase, sustained effect of COVID-19 pandemic, immediate effect of tobacco tax increase, immediate effect of COVID-19 pandemic each indicates coefficient α_2_, α_3_, α_4_, and α_5_ calculated by the interrupted time series analysis.
